# Rare Earth Elements in Human Calcified Aortic Valves and Epicardial Adipose Tissue

**DOI:** 10.3390/jcm14092891

**Published:** 2025-04-22

**Authors:** Barbara Poniedziałek, Bartłomiej Perek, Aleksandra Proch, Marcin Misterski, Anna Komosa, Przemysław Niedzielski, Andrzej Fal, Marek Jemielity, Piotr Rzymski

**Affiliations:** 1 Department of Environmental Medicine, Poznan University of Medical Sciences, 60-806 Poznań, Poland; bpon@ump.edu.pl; 2 First Department of Cardiac Surgery and Transplantology, Poznan University of Medical Sciences, 61-848 Poznań, Poland; bperek@ump.edu.pl (B.P.); mmisterski@ump.edu.pl (M.M.); kardiock@ump.edu.pl (M.J.); 3 Department of Analytical Chemistry, Adam Mickiewicz University, 61-614 Poznań, Poland; aleksandra.proch@amu.edu.pl (A.P.); pnied@amu.edu.pl (P.N.); 4 Department of Clinical Pharmacology, Poznan University of Medical Sciences, 61-848 Poznań, Poland; komosa.ania@ump.edu.pl; 5 Collegium Medicum, Cardinal Wyszynski University, 02-507 Warsaw, Poland; a.fal@uksw.edu.pl; 6 National Medical Institute of the Ministry of the Interior and Administration, 02-507 Warsaw, Poland

**Keywords:** emerging pollutants, aortic stenosis, chemical analyses, human tissue, cardiovascular system

## Abstract

**Background/Objectives:** Rare earth elements (REEs) are emerging environmental pollutants, with human exposure increasing due to recent industrial and technological activities. While most studies have focused on detecting REEs in human fluids, their presence in tissues remains understudied. Aortic valve degeneration is known to facilitate the adsorption of various chemical elements; however, the occurrence of REEs in human valves has not yet been investigated. This exploratory study aimed to determine the presence of REEs in the aortic valves of patients with aortic stenosis undergoing surgical valve replacement. It also analyzed potential correlations between REE levels in the valves, epicardial adipose tissue, serum, and selected disease markers. **Methods:** Samples of aortic valve, epicardial adipose tissue, and serum were collected from 20 adult patients undergoing elective aortic valve replacement. The concentrations of 14 REEs in these samples were measured using inductively coupled plasma mass spectrometry. Biochemical and clinical parameters of the patients were also considered to explore potential associations with the determined REE levels. **Results:** Total REEs, heavy REEs, and light REEs in aortic valves, epicardial fat, and serum were not intercorrelated. Moreover, for any sample type, they were not significantly related to the patient’s demographics (age and sex), clinical characteristics (body mass index, heart failure severity, and systolic pressure gradients), kidney function (estimated glomerular filtration rate), and biochemical markers (creatinine, lipoprotein(a), total cholesterol, HDL, LDL, and fibrinogen). Smoking was the only factor influencing REE burden in studied patients, with active smokers revealing 61% higher serum REE concentrations and past smokers exhibiting 133% higher REE valvular deposition. **Conclusions:** The findings suggest that REE accumulation in aortic valve tissues occurs independently of systemic and clinical parameters but may be promoted by smoking, highlighting the need to investigate the underlying mechanisms of REE deposition. Given the small sample size and the cross-sectional, hypothesis-generating design, these observations should be interpreted with caution and treated as preliminary. Larger, longitudinal studies are needed to validate these results and explore potential causal relationships. Further research should also include the tissue originating from individuals without aortic stenosis for comparison. A deeper understanding of the pathways and health risks associated with REEs in cardiovascular tissues may offer valuable insights into their broader implications for human health.

## 1. Introduction

Rare earth elements (REEs) consist of seventeen chemical elements, namely lanthanum (La), cerium (Ce), europium (Eu), gadolinium (Gd), neodymium (Nd), praseodymium (Pr), promethium (Pm), samarium (Sm), and scandium (Sc), which are categorized as light REEs (LREEs), and dysprosium (Dy), erbium (Er), holmium (Ho), lutetium (Lu), terbium (Tb), thulium (Tm), yttrium (Y), and ytterbium (Yb), which represent heavy REEs (HREEs) [1]. These are employed in a growing number of industrial, medical, and agricultural applications and remain critical for advanced technologies like electronics, renewable energy systems, and defense applications [2]. Their industrial extraction, refining, and disposal processes often release REEs into air, soil, and water systems, posing risks to ecosystems and human health [3,4]. These non-essential elements, though typically present in trace amounts, can accumulate in the environment and organisms [5,6,7,8]. Moreover, their persistence and bioavailability raise concerns about long-term contamination and exposure. As demand for REEs grows, it is imperative to develop sustainable management practices and remediation technologies to mitigate their environmental impact [2,9].

Routes of human exposure to REEs include the inhalation of dust or aerosols during mining and processing as well as the consumption of contaminated water or food [10,11]. REEs have been detected in various human fluids and tissues, raising concerns about their potential health implications. Trace levels of REEs have been found in blood, urine [12,13,14], sperm [15], breast milk [16], and bone tissue, often as a result of environmental exposure or occupational hazards. Particularly high levels of REEs in humans were noted in mining areas [17,18,19]. While the exact biological effects of chronic REE accumulation are not fully understood, preliminary studies suggest that they can potentially induce oxidative stress, DNA damage, and cell death [20] and that the clinical outcomes include an increased risk of hypertension [21], myocardial infarction [22], and pregnancy failure [23].

Aortic valves can act as an accumulation site for various trace elements, particularly when they degenerate and become calcified. Calcification primarily involves the deposition of calcium and phosphate, forming hydroxyapatite crystals. It is known that, apart from passive diffusion, valvular mineralization involves complex biophysical and biochemical processes, including tissue remodeling, oxidative stress, inflammation, hydroxyapatite’s ability to adsorb chemical elements, and the overexpression of metalloproteins in diseased valves [24,25]. Previous studies have shown some association between trace element accumulation and aortic valve degeneration [26,27]. However, no data currently exist on the presence or implications of REEs in calcified aortic valves, representing a significant knowledge gap. This is also true for epicardial adipose tissue, which is located between the myocardium and the visceral layer of the pericardium, and which, due to the direct anatomic and functional proximity to the myocardium and coronary arteries, can potentially influence the progression of cardiovascular pathologies, including aortic stenosis. It has been increasingly recognized as a metabolically active tissue capable of influencing cardiovascular diseases [28] through the secretion of various factors, including adiponectin, adrenomedullin, adipokines, several proinflammatory cytokines, monocyte chemoattractant protein-1, nerve growth factor, resistin, plasminogen activator inhibitor-1, and free fatty acids [29].

Investigating their potential accumulation could expand the understanding of how modern environmental exposures intersect with cardiovascular pathology and potentially offer novel insights into the environmental and biochemical factors contributing to valvular disease. This is important since the burden of aortic stenosis is already substantial and generates high economic costs due to the increasing number of valve prostheses annually implanted worldwide [30,31], while the number of patients requiring surgical interventions for degenerated valves is projected to double by 2050 [32].

Therefore, this exploratory study aimed to determine the content of REEs in aortic valves resected during valve replacement surgery performed in patients with aortic stenosis. The REE levels were also assessed in the patient’s serum and epicardial adipose tissue to understand whether valvular concentrations reflect systemic accumulation or localized deposition driven by calcification processes. This approach could help elucidate whether REEs actively contribute to calcific aortic valve disease or simply accumulate passively in diseased tissues. Moreover, the relationship of REE burden with patient clinical characteristics, echocardiographic examination, and biochemical parameters was also explored to evaluate potential correlations between environmental exposure, systemic health status, and cardiovascular pathology. Identifying such associations could provide valuable insights into a better understanding of how REE exposure and accumulation can contribute to disease in humans.

## 2. Materials and Methods

### 2.1. Patient Recruitment

This study enrolled 20 adult patients undergoing elective surgical aortic valve replacement, performed at a single experienced cardiac surgery department (a volume of more than 1000 surgical procedures per year) either as isolated procedures (50%) or combined with other interventions (10% with coronary artery bypass grafting, 35% with ascending aortic replacement, and 5% with mitral valve replacement). Eligibility for surgery was determined through comprehensive clinical and imaging evaluations, including echocardiography, coronary angiography, and computed tomographic angiography if indicated. All surgeries were conducted under general anesthesia from partial or complete median sternotomy using standard cardiopulmonary bypass techniques with cold crystalloid cardioplegic myocardial arrest.

### 2.2. Sample Collection

During surgery, degenerated aortic cusps were quickly excised, followed by aortic cross-clamping, the induction of cardioplegic cardiac arrest, and the exposure of the aortic valve through aortotomy. Surgeons paid special attention to performing the complete debridement of both aortic valve and annulus. These valve samples were rinsed thoroughly with ultrapure Milli-Q water (Millipore, Darmstadt, Germany) to remove blood residues and stored in trace element-free cryogenic tubes at −40 °C until further analysis. Serum samples were preserved under identical conditions. Blood samples were collected upon the patient’s arrival in the operating theater before surgery. After clotting, these samples were centrifuged at 2000× *g* for 10 min to isolate serum. Additional blood samples were collected for biochemical marker analysis in the in-hospital accredited diagnostic laboratory.

### 2.3. Sample Mineralization

Prior to analysis, all samples were thawed, weighed, and digested using concentrated 65% HNO_3_ (Sigma-Aldrich, Darmstadt, Germany) in closed Teflon containers within a Mars 6 Xpress microwave digestion system (CEM Corporation, Matthews, NC, USA), similarly to previous studies focusing on REE determination in human samples [16,33]. The digestion protocol involved heating to 180 °C over 20 min, followed by a 30 min hold at that temperature. Serum samples (1 mL) were digested in 3 mL of 65% HNO_3_. After mineralization, samples were filtered and diluted with Milli-Q water, yielding final volumes of 15.0 mL for valve samples and 5.0 mL for serum samples.

### 2.4. Determination of Rare Earth Elements

The levels of fourteen REEs (Ce, Eu, Er, La, Gd, Nd, Pr, Sm, Dy, Ho, Lu, Tb, Tm, and Yb) in aortic valves, epicardial adipose tissue, and serum were determined with a PlasmaQuant MS Q inductively coupled plasma mass spectrometer (Analytik Jena, Jena, Germany). The instrument was configured with the following parameters: nebulizer gas flow at 0.98 L/min; plasma gas flow at 9.0 L/min; auxiliary gas flow at 1.5 L/min; and radio frequency power set to 1.20 kW. Measurements were taken in 5 replicates, with 20 scans per replicate. Interference reduction was facilitated by the integrated collision reaction cell, which sequentially operated in three modes: without gas, with helium as the collision gas, and with hydrogen as the reaction gas. For the detection of REEs, the helium mode was employed at a gas flow rate of 80 mL/min. Sensitivity was optimized by utilizing the reflexION ion mirror, broadening the detectable concentration range. The instrument’s detection limits were as follows: Ce—0.0013 µg/L, Eu—0.003 µg/L, Er—0.0003 µg/L, La—0.0092 µg/L, Gd—0.007 µg/L, Nd—0.0025 µg/L, Pr—0.0005 µg/L, Sm—0.009 µg/L, Dy—0.005 µg/L, Ho—0.011 µg/L, Lu—0.011 µg/L, Tb—0.0001 µg/L, Tm—0.01 µg/L, and Yb—0.0005 µg/L. The analytical procedure was validated against a wide set of several certified reference materials, including ERM-DA-120a (human serum), DB001 (human hair), BCR-185R (bovine liver), ERM-BB184 (bovine muscle), BCR-668 (mussel tissue), Seronorm^Tm^-L1 (human urine), and BCR-627 (tuna fish tissue). Quality assurance involved the use of ERM-BB184 bovine muscle (certified by the European Commission Joint Research Centre, Geel, Belgium) and the standard addition method. Recovery rates between 80% and 120% were considered satisfactory.

### 2.5. Statistical Analyses

Statistical analyses were performed using Statistica v.13.3 (StatSoft, USA). The non-parametric approach was employed due to a low sample size and data not adhering to a Gaussian distribution. REE levels between the two groups were compared with the Mann–Whitney U test, while Spearman’s rank correlation coefficient (Rs) was used to assess the association of REE contents with the biochemical, clinical, and demographic parameters of patients. Graphs, including heatmaps, were drawn with GraphPad Prism 8.0.1 (GraphPad Software, San Diego, CA, USA). A *p*-value below 0.05 was considered statistically significant in all analyses.

### 2.6. Ethical Approval

The study protocol adhered to the ethical guidelines established by the 1964 Declaration of Helsinki and its subsequent revisions. Approval was obtained from the Local Bioethical Committee at the Poznan University of Medical Sciences, Poznan, Poland (approval number 518/23, issued on 29 June 2024). Written informed consent was obtained from all participants prior to inclusion.

## 3. Results

### 3.1. Studied Patients

The demographical and clinical characteristics of the studied group of patients with aortic stenosis (*n* = 20) are outlined in Table 1. The cohort consisted of individuals with an average age of 68.9 years, equally distributed above and below 70 years. Males comprised the majority of the studied population. Obesity was prevalent in 45% of participants, with a mean BMI of 29.4 kg/m^2^. Biochemical assessments indicated average creatinine levels at 85.6 µmol/L and total cholesterol levels of 3.7 mmol/L. Preoperative echocardiography revealed elevated mean systolic pressure gradients in 35% of cases. Two patients had a bicuspid aortic valve diagnosed in the preoperative echocardiography that was confirmed intraoperatively.

The most prevalent comorbidities were arterial hypertension (85%, *n* = 17), diabetes mellitus (45%, *n* = 9), coronary artery disease (35%, *n* = 7), and ascending aortic aneurysms (35%, *n* = 7). Moreover, half of the patients were found in classes III or IV of the New York Heart Association (NYHA) functional classification, with the rest in class II. Of note, none of the studied patients had a history of the use of Gd-based contrast agents in magnetic resonance imaging or magnetic resonance angiography, and none had an occupational risk of exposure to REEs through mining activities or work at a factory employing REEs in the production process.

### 3.2. Levels of Rare Earth Elements in Aortic Valves and Epicardial Tissue

**Figure 1:**
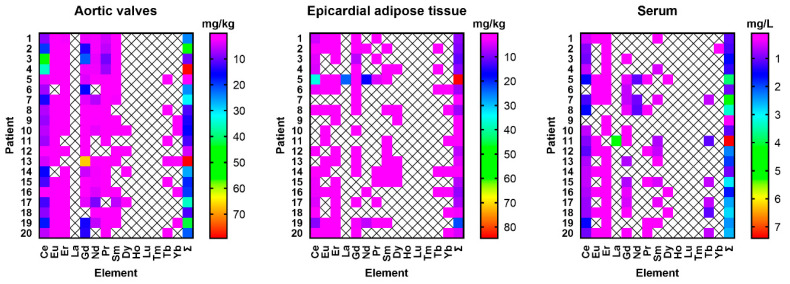
The distribution of rare earth elements in the aortic valves, epicardial adipose tissue, and serum of patients with aortic stenosis. X represents concentrations below the level of detection.

The levels of particular REEs in the analyzed aortic valves, epicardial adipose tissue, and serum of patients with aortic stenosis are summarized in Table 2. Light REEs were the dominant fraction in all samples, representing 95.5% of all REEs found in aortic valves, 89.1% in epicardial fat, and 78.3% in serum. However, a distinct distribution pattern across the three sample types was observed (Figure 1). Aortic valves exhibited the highest total REE contents, with notable levels of Ce and Gd. Epi. Epicardial adipose tissue revealed lower levels of REEs, with Nd and Ce being particularly prominent. The serum REE concentration was low, with the highest levels noted for Ce, Tb, and Nd. Ho, Lu, and Tm were below the detection levels in all sample types (Figure 1), while La was only determined in a single sample of epicardial fat tissue.

### 3.3. Association Between Rare Earth Elements Levels and Demographic, Biochemical, and Clinical Parameters

**Figure 2:**
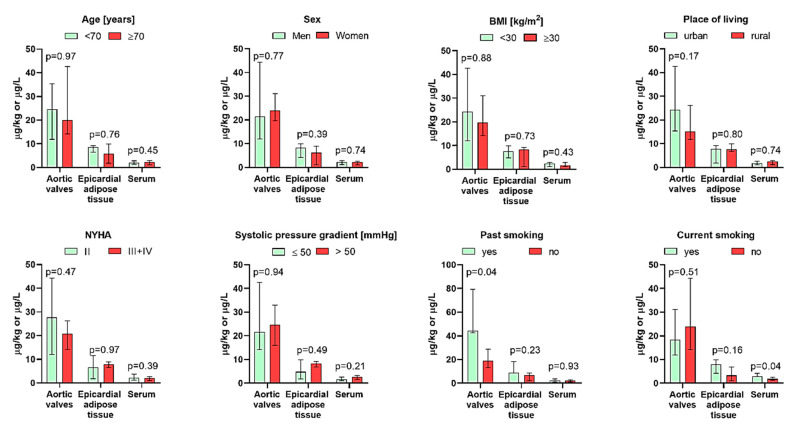
Association between levels of rare earth elements in the aortic valves, epicardial adipose tissue, serum, and other characteristics of patients with aortic stenosis (*n* = 20). Bars represent the median, and whiskers represent the interquartile range.

**Figure 3:**
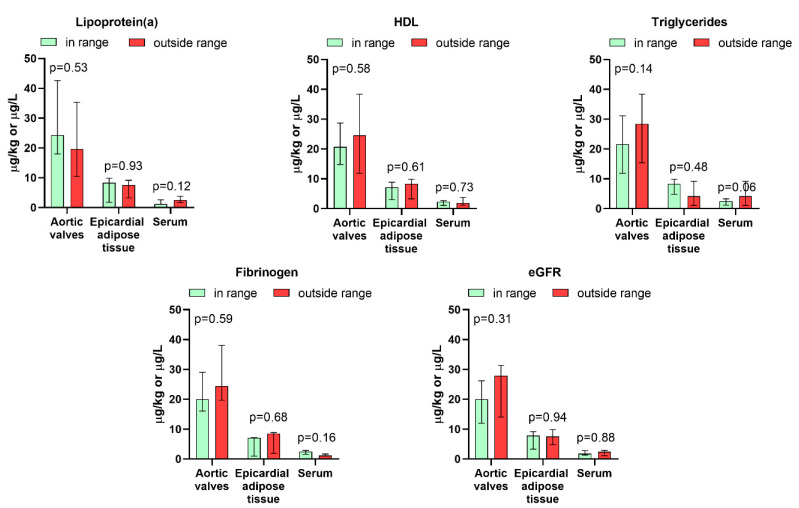
Association between levels of rare earth elements in the aortic valves, epicardial adipose tissue, serum, and serum biochemistry of patients with aortic stenosis (*n* = 20). Bars represent the median, and whiskers represent the interquartile range.

No significant intercorrelations were observed between the total REE, light REE, and heavy REE levels in the aortic valves, epicardial adipose tissue, and serum (Table 3). Furthermore, the REE content in any type of sample did not correlate with the patient’s age or BMI, did not differ between men and women or obese and non-obese individuals (Table 4, Figure 2), and was not significantly associated with any biochemical serum parameter considered as risk factors for the development of calcified aortic valve degeneration, namely creatinine, total cholesterol, LDL, HDL, triglycerides, fibrinogen, and lipoprotein(a), as well as eGFR (Table 4, Figure 3). Moreover, it was not correlated with the mean and peak systolic pressure gradients (Table 4), did not differ between individuals with the former parameter exceeding and not exceeding 50 mmHg, and was not differentiated between patients having NYHA class 3 + compared to those scoring 2 (Figure 2). Active smokers had 61% higher concentrations of REEs in serum, while previous smokers had 133% higher valvular REE contents. No differences in the levels of these elements were found between inhabitants of rural and urban areas (Figure 2).

## 4. Discussion

This exploratory study provides novel information on the REE content in human aortic valves originating from patients with severe aortic stenosis, in whom degenerated and calcified valves are known to accumulate various trace metals, fueling inflammation and further disease progression. It is also the first to report on the content of these elements in epicardial adipose tissue and to seek associations between the accumulated and circulating REE levels and various clinical and biochemical markers in aortic stenosis.

The study shows that the valvular content was low compared to the levels of numerous other trace elements determined in calcified aortic valves in other research [26,34,35]. The total REE level was in the range noted previously for Co and Cr [26].

Moreover, our work did not detect any correlations between the REE levels in valves and serum. This is not an unexpected finding since the accumulation of chemical elements in aortic valves is not subject to a simple passive deposition but is suggested to result from a combination of various cellular and biochemical processes, including mineralization, tissue remodeling, oxidative stress, inflammation, the overexpression of metalloproteins, and adsorption on hydroxyapatite [25,36]. In addition, it must be stressed that the development of calcific aortic valve stenosis is a long-lasting process, and thus, the accumulation of any elements may be the effect of many years of environmental exposure. Although no direct correlation was observed between the circulating and valvular REE levels in our cohort, this does not necessarily undermine the potential utility of serum levels for biomonitoring or clinical follow-up. Rather, it may indicate the need to establish specific reference ranges or thresholds for different biological matrices. Since serum and urine are, along with hair, the most frequently employed human samples for the biomonitoring of REE exposure [13,14,37,38,39,40], our work underlines that the analysis of such material may not necessarily reflect the burden and accumulation of REEs in tissues. Nevertheless, further research is warranted to determine whether serum REE levels could serve as early indicators of exposure or dynamic changes, even in the absence of the direct mirroring of tissue contents. Therefore, further studies should be performed exploring the levels of these elements in various other human tissues that are known to accumulate trace metals and that can be collected, along with serum, during routine diagnostic or therapeutic procedures from living individuals [41,42]. This would advance the understanding of REE distribution in the human body in relation to their circulating levels.

Previous research has evidenced that age, sex, and BMI can all differentiate the accumulation of various trace metals in different human tissues [43,44,45]. Recently, this has also been shown for aortic valves. For example, women have revealed lower valvular contents of Ca, Mg, P, Pb, and Zn, but higher levels of Cd, Co, S, Se, and Sn. Moreover, in healthy valves, the contents of Ba, Ca, Co, P, Pb, and Sr increased with age [26]. Although the referenced study did not investigate REE contents, other research has demonstrated that the accumulation of these elements in the human rib bone tissue is age-dependent [46]. Such a phenomenon was also shown in adult hair, especially in the case of Ce [39]. However, in our study of patients with aortic stenosis, none of the parameters discussed were related to differences in the REE levels in aortic valves, epicardial adipose tissue, and serum.

This implies that other factors, including lifestyle-related factors, may potentially play a role in the presence of REEs in the studied cohort of patients. Cigarette smoking has been recognized as a potential source of REE exposure since all of these elements were determined in both tobacco and rolling paper [47], while increased concentrations of Ce and La were found in indoor air due to environmental tobacco smoke [48], and REEs were more frequently detected in the serum of smokers compared to non-smokers [49]. Therefore, in the present study, we have explored whether cigarette smoking can affect the levels of these chemicals found in patients with aortic stenosis. As demonstrated, active smokers had significantly higher serum REEs, while past smokers had increased the valvular deposition of these elements. These findings are of high interest, especially if one considers that smoking is an established risk factor for the development of cardiovascular diseases and has been shown to accelerate the progression of aortic stenosis [50,51,52].

Beyond smoking, non-occupational exposure to rare earth elements (REEs) in the studied cohort may also stem from dietary sources. In Poland, wild edible mushrooms, commonly harvested from forests, have been shown to accumulate REEs in their fruiting bodies [5,53]. Additionally, certain dietary supplements available in the Polish market contain elevated REE levels, though data on these products remain limited [54]. Other potential dietary sources include vegetables and other foodstuffs, though further research is needed to assess their contribution [55]. Airborne exposure may also play a role, as poor air quality in Poland, driven by emissions of particulate matter from combustion processes [56], could introduce REEs into the environment [57]. While evidence is still emerging, this suggests another possible pathway for general population exposure. Nevertheless, more research into routes of REE exposure in Poland is necessary to better understand the main pathways.

We have also explored whether REE levels in serum, aortic valves, and epicardial adipose tissue can be related to various biochemical parameters. Previous research has found that REE exposure can be associated with lower serum triglyceride levels and increased cholesterol concentrations, suggesting that REEs might influence lipid metabolism [58]. However, no correlations between these parameters and REEs in the analyzed sample types were found in our group of patients with aortic stenosis. This discrepancy could be attributed to differences in exposure levels, population characteristics, or the pathophysiological state of our cohort, which may override or obscure potential associations. Further, no other biochemical parameter considered in our study (creatinine, lipoprotein(a), HDL, LDL, and fibrinogen) revealed any association with REEs. This lack of correlation might indicate that REEs do not have a direct or substantial influence on these markers in the context of aortic stenosis. Alternatively, any potential effects of REEs on these parameters may be subtle and thus undetectable in the presence of the pronounced biochemical and metabolic changes characteristic of advanced cardiovascular disease [59,60].

Although the levels of rare earth elements (REEs) detected in the aortic valves were relatively low, their presence in cardiovascular tissues may still carry clinical significance. For example, Pr has experimentally been shown to alter cardiovascular development [61]. Moreover, exposure to REEs has been shown in experimental studies to induce oxidative stress, apoptosis, and inflammatory signaling pathways [62], all of which are involved in the pathogenesis of aortic valve calcification and stenosis. Moreover, REEs have a similar ionic radius to calcium, so they may potentially interfere with its regulation [20]. While our study did not identify specific clinical or biochemical correlations with REE levels, the bioactivity of these elements warrants further mechanistic investigation, particularly in the context of chronic cardiovascular diseases characterized by inflammation and progressive tissue remodeling. Clarifying the role of REEs in these processes could ultimately have implications for both understanding disease progression and identifying environmental or lifestyle factors that may exacerbate cardiovascular pathology.

Although this study provides novel insights into the presence of REEs in humans, its limitations must also be stressed. Multicenter studies would be beneficial to confirm these observations and explore variability across broader populations, including groups with occupational exposure to REEs or living in areas with increased levels of pollution by these elements. The present study was based on a small sample size, with some groups represented by a low number of individuals (e.g., active smokers). Moreover, the cross-sectional design precludes establishing causal relationships between REE exposure and their accumulation in aortic valves. Longitudinal studies tracking REE levels in patients with progressive aortic stenosis are warranted to determine temporal patterns of deposition and potential clinical implications. Additionally, the analysis was conducted on samples originating exclusively from patients undergoing valve replacement for severe aortic valve calcific degeneration, which may not represent the REE dynamics in individuals with healthy, non-calcified valves. As such, the absence of a validation cohort, including patients with early-stage or non-degenerated valves, limits our ability to assess whether REE accumulation is a contributing factor in disease initiation or is a consequence of advanced degeneration. Future studies incorporating such control groups will be essential to elucidate the potential role of REEs in the pathogenesis of aortic stenosis.

## 5. Conclusions

This exploratory study provides the first evaluation of REEs in human aortic valves, epicardial adipose tissue, and serum, specifically in patients with severe aortic stenosis. The findings demonstrate that the REE content in calcified aortic valves is relatively low compared to other trace elements, though higher than in epicardial adipose tissue, and is not correlated with their circulating levels in serum. These results suggest that REE accumulation in aortic valves likely arises from complex cellular and biochemical processes rather than passive deposition. It also highlights the limitations of relying solely on serum or urine analyses for assessing tissue REEs accumulation and underscores the need for further investigations into REE distribution in various human tissues. No associations were observed between REE levels and clinical or biochemical parameters in the studied cohort. However, subtle effects or interactions may remain undetected within the advanced pathophysiological state of this disease. Smoking was found to be the only factor influencing the REE burden in studied patients, with active smokers revealing higher serum REE concentrations and past smokers exhibiting increased REE valvular deposition. However, due to the small sample size, including a low number of smokers, the reported associations should be interpreted as preliminary and with appropriate caution and require validation in larger, more powered cohorts. Longitudinal and multicenter studies are recommended to explore temporal patterns of REE deposition, assess variability across diverse populations, and clarify potential health implications. Additionally, future research should include individuals with healthy valves to better understand REE dynamics in non-calcified tissues. These efforts will contribute to a more comprehensive understanding of REE biology and the role of REEs in human health and disease.

## Figures and Tables

**Table 1:** Characteristics of the studied group of patients with aortic stenosis (*n* = 20) from whom the aortic valve, epicardial adipose tissue, and serum samples were collected and analyzed.

Demographic Data	Mean ± SD or % (*n*)
Age (years)	68.9 ± 7.4
<70 years	50 (10)
≥70 years	50 (10)
Male/Female	70/30 (14/6)
Body Mass Index (BMI, kg/m^2^)	29.4 ± 4.6
Obesity, BMI > 30 kg/m^2^	45 (9)
Rural/urban inhabitants	30/70 (6/14)
Current smokers (within last year)	15 (3)
Past smokers (cessation within 1–10 years)	25 (5)
Biochemical parameters	**mean ± SD**
eGFR (mL/min/1.73 m^2^) in reference range—≥90 mL/min/1.73 m^2^ (%, (*n*))	86.4 ± 25.9 50 (10)
Creatinine (µmol/L) in reference range—53–115 µmol/L (%, (*n*))	85.6 ± 17.3 90 (18)
Total cholesterol (mmol/L) in reference range—5.0 mmol/L (%, (*n*))	3.7 ± 1.1 85 (17)
Low-density lipoprotein (mmol/L) in reference range, <2.6 mmol/L, (%, (*n*))	1.9 ± 0.9 80 (16)
High-density lipoprotein (mmol/L) in reference range—≥1.3 mmol/L, (%, (*n*))	1.4 ± 0.4 60 (12)
Triglycerides (mmol/L) in reference range, <1.7 mmol/L (%, (*n*))	1.1 ± 0.4 75 (15)
Fibrinogen (mg/dL) in reference range—200–400 mg/dL (%, (*n*))	356.5 ± 84.0 70 (14)
Lipoprotein (mg/dL) in reference range, <30 mg/dL (%, (*n*))	40.8 ± 51.1 55 (11)
Preoperative Echocardiographic Findings (M+2D+Doppler)	**mean ± SD or % (** ***n*** **)**
Mean systolic pressure gradient (mmHg) >50 mmHg (%, (*n*))	53.9 ± 15.9 35.0 (7)
Peak systolic pressure gradient (mmHg)	82.3 ± 23.9
Bicuspid aortic valve (%, (*n*))	10.0 (2)

**Table 2:** Rare earth elements (REEs) in the aortic valves, epicardial fat, and serum of patients with aortic stenosis (*n* =20).

Element	Aortic Valves [µg/kg]		Epicardial Fat [µg/kg]		SERUM [µg/L]	
	>LOD (*n*)	Mean ± SD (max)	>LOD (*n*)	Mean ± SD (max)	>LOD (*n*)	Mean ± SD (max)
Ce	20	12.9 ± 12.4 (55.1)	15	5.9 ± 8.5 (35.9)	18	0.9 ± 0.03 (1.5)
Eu	18	0.6 ± 0.3 (1.1)	12	0.4 ± 0.1 (0.6)	10	0.2 ± 0.01 (0.2)
Er	18	0.8 ± 0.6 (2.7)	15	0.5 ± 0.2 (1.0)	18	0.2 ± 0.1 (0.3)
La	0	-	1	23.4	1	4.3
Gd	19	10.6 ± 15.9 (66.6)	15	1.9 ± 1.4 (6.6)	13	0.4 ± 0.2 (0.7)
Nd	16	3.5 ± 2.0 (9.2)	4	6.9 ± 6.5 (16.4)	3	1.1 ± 0.1 (1.1)
Pr	18	2.3 ± 2.8 (9.7)	7	1.2 ± 1.5 (4.5)	6	0.2 ± 0.1 (0.3)
Sm	15	1.3 ± 1.4 (5.9)	10	1.5 ± 0.5 (2.5)	9	0.4 ± 0.1 (0.7)
Dy	4	0.9 ± 0.6 (1.8)	6	0.7 ± 0.2 (1.1)	3	0.2 ± 0.1 (0.3)
Ho	0	-	0	-	0	-
Lu	0	-	0	-	0	-
Tb	6	1.3 ± 0.8 (2.6)	5	1.6 ± 0.1 (1.7)	6	0.8 ± 0.3 (1.1)
Tm	0	-	0	-	0	-
Yb	5	0.1 ± 0.2 (0.7)	7	0.7 ± 0.2 (1.0)	1	0.2
Σ Light REEs	20	29.5 ± 24.7 (90.1)	20	9.8 ± 18.2 (84.3)	20	1.8 ± 1.4 (6.3)
Σ Heavy REEs	20	1.4 ± 0.8 (3.3)	20	1.2 ± 1.1 (3.7)	20	0.5 ± 0.4 (1.5)
Σ Total REEs	20	30.9 ± 24.8 (90.9)	20	11.0 ± 18.0 (84.6)	20	2.3 ± 1.6 (7.4)

**Table 3:** Correlation matrix (Spearman’s correlation coefficient) between levels of rare earth elements in the aortic valves, epicardial adipose tissue, and serum of patients with aortic stenosis (*n* = 20).

Total REEs		
	Epicardial adipose tissue	Serum
Aortic valves	0.31 (*p* = 0.19)	−0.32 (*p* = 0.16)
Epicardial adipose tissue	-	−0.16 (*p* = 0.49)
Light REEs		
	Epicardial adipose tissue	Serum
Aortic valves	0.33 (*p* = 0.14)	−0.33 (*p* = 0.15)
Epicardial adipose tissue	-	0.02 (*p* = 0.92)
Heavy REEs		
	Epicardial adipose tissue	Serum
Aortic valves	−0.25 (*p* = 0.29)	0.08 (*p* = 0.75)
Epicardial adipose tissue	-	−0.22 (*p* = 0.36)

**Table 4:** Correlations (Spearman’s correlation coefficient; Rs) between the total content of rare earth elements (REEs) in the aortic valves, epicardial adipose, serum, and various other characteristics of patients with aortic stenosis (*n* = 20).

Parameter	Aortic Valves		Epicardial Adipose Tissue		Serum	
	*Rs*	*p*	*Rs*	*p*	*Rs*	*p*
Age	−0.04	0.88	−0.11	0.64	−0.13	0.60
BMI	0.06	0.81	0.05	0.83	−0.21	0.81
Creatinine	−0.22	0.36	0.03	0.91	−0.04	0.85
Total cholesterol	−0.01	0.95	−0.08	0.74	−0.28	0.24
LDL	−0.06	0.81	−0.08	0.74	−0.31	0.19
HDL	−0.08	0.75	−0.12	0.61	−0.00	0.99
Triglycerides	0.32	0.18	−0.12	0.63	−0.29	0.23
Lipoprotein(a)	−0.07	0.77	−0.05	0.85	0.00	0.99
Fibrinogen	0.32	0.16	0.02	0.93	−0.24	0.30
eGFR	0.13	0.59	−0.02	0.93	−0.13	0.59

## Data Availability

The datasets used and analyzed during the current study are available from the corresponding author upon reasonable request.
